# Replication-Dependent and Transcription-Dependent Mechanisms of DNA Double-Strand Break Induction by the Topoisomerase 2-Targeting Drug Etoposide

**DOI:** 10.1371/journal.pone.0079202

**Published:** 2013-11-07

**Authors:** Margaret Tammaro, Peri Barr, Brett Ricci, Hong Yan

**Affiliations:** Fox Chase Cancer Center, Philadelphia, Pennsylvania, United States of America; University of Minnesota, United States of America

## Abstract

Etoposide is a DNA topoisomerase 2-targeting drug widely used for the treatment of cancer. The cytoxicity of etoposide correlates with the generation of DNA double-strand breaks (DSBs), but the mechanism of how it induces DSBs in cells is still poorly understood. Catalytically, etoposide inhibits the re-ligation reaction of Top2 after it nicks the two strands of DNA, trapping it in a cleavable complex consisting of two Top2 subunits covalently linked to the 5’ ends of DNA (Top2cc). Top2cc is not directly recognized as a true DSB by cells because the two subunits interact strongly with each other to hold the two ends of DNA together. In this study we have investigated the cellular mechanisms that convert Top2ccs into true DSBs. Our data suggest that there are two mechanisms, one dependent on active replication and the other dependent on proteolysis and transcription. The relative contribution of each mechanism is affected by the concentration of etoposide. We also find that Top2α is the major isoform mediating the replication-dependent mechanism and both Top2α and Top2 mediate the transcription-dependent mechanism. These findings are potentially of great significance to the improvement of etoposide’s efficacy in cancer therapy.

## Introduction

Etoposide (VP16) is one of the most widely used drugs for the treatment of various types of human malignancy, including leukemia, lymphoma, and solid tumors [1–4]. However, its efficacy varies significantly among different types of cancer. In addition, it is associated with the serious side effect of secondary leukemia resulting from drug induced chromosome translocations [5,6]. The cytotoxicity and the side effects of etoposide are both correlated with the induction of DNA double-strand breaks in cells [7,8]. Better understanding of how etoposide induces DSBs and their repair is of great significance to the maximization of the therapeutic efficacy as well as the minimization of the side effects of this important drug.

The primary cellular target of etoposide is DNA topoisomerase 2 (Top2), a homodimeric enzyme that changes the topology of DNA [2,3]. Mammalian cells contain two Top2 isoforms, Top2α and Top2β, which share ca. 70% sequence identity [9–12]. Top2α is highly expressed in dividing cells and tumor cells, and further up-regulated during S and G2 phases [13–15]. It is essential for cell proliferation, participating in replication, transcription, and chromosome structure and segregation [16]. Top2β is expressed in dividing as well as non-dividing cells [17]. It is dispensable for cell proliferation, but required for development (Top2β knockout mice die from neural defect at birth) and appears to participate in transcription [18–20]. Catalytically, the two isoforms use the same mechanism and are inhibited indiscriminately by etoposide and thus often collectively referred to as Top2 [21,22]. During the catalytic cycle, each subunit of Top2 nicks one strand of DNA to generate a double-strand break, through which another DNA then passes, resulting in changes of topology [23]. The 5’ end of each nick is covalently linked to a tyrosine residue at the catalytic center of each subunit, forming a Top2-DNA cleavable complex (Top2cc). The 3’ ends are juxtaposed to the 5’ ends, allowing the nicks to be religated after the passage of the target strand to complete the catalytic cycle. Upon binding of etoposide, Top2 is trapped at the Top2cc intermediate step [23,24]. However, the two sides of the DSB are still held together by the strong interaction between the two subunits of Top2 and will be immediately resealed once etoposide has dissociated [25]. For Top2cc to be recognized as a true DSB, it has to be further processed by cells [2]. Despite its importance, the mechanism by which cells convert a Top2cc into a true DSB is still not well understood.

Top2cc is expected to be directly sensed as a roadblock to the progression of replication and transcription machineries. It has been observed that transcription stimulates the degradation of etoposide-trapped Top2ccs. Trapped Top2 is ubiquitinated and then degraded by the 26S proteosome [26]. The ubiquitination step is independent of transcription, but the degradation step is strongly stimulated by transcription [27]. In principle, the degradation of Top2cc should convert a Top2cc into a true DSB. In support of this hypothesis, inhibitors of either transcription or the 26S proteosome cause significant reductions in the number of etoposide-induced DSBs based on neutral COMET assays [28]. Both isoforms of Top2 are degraded after etoposide treatment, but Top2β is degraded much more rapidly and extensively than Top2α [29]. In line with this difference, Top2β has been suggested to be the dominant isoform mediating the transcription-dependent DSB induction by etoposide [30,31]. However, it has also been shown by many (though not all) studies that Top2α rather than Top2β is the dominant isoform mediating cytoxicity of etoposide in human cells [30,32] and in mice [33]. This apparent paradox suggests that the transcription-dependent mechanism might not be the only one for DSB induction by etoposide. The most logical alternative mechanism is through replication, which in principle should also collide with Top2cc and might result in DSB formation. This has been implied by observations that inhibiting replication can partially rescue the cytotoxicity of etoposide [34,35]. However, there has been no direct evidence for this mechanism of DSB induction by etoposide or other Top2-targeting drugs.

In this study we have investigated the cellular mechanisms by which etoposide induces DSBs in cells. Our data revealed that there are two mechanisms, one mechanism dependent on DNA replication and the other on transcription. The transcription-dependent mechanism requires proteolysis, but the replication-dependent mechanism does not. The relative contribution of each mechanism is affected by the concentration of etoposide. At low concentrations of etoposide, the replication-dependent mechanism dominates, whereas at high concentrations of etoposide, both mechanisms are active. We also found that Top2α and Top2β can both mediate DSB induction. Top2α is the major isoform responsible for the replication-dependent mechanism, and Top2α and Top2β are both capable of mediating the transcription-dependent mechanism. These findings are of great significance to the understanding of the generation and repair mechanisms of etoposide-induced DSBs and the improvement of etoposide efficacy in cancer therapy. 

## Materials and Methods

### Cell culture and reagents

The human osteosarcoma (U2OS) cells, Dulbecco's Modified Eagle Medium (DMEM), fetal bovine serum, penicillin/streptomycin (P/S), L-glutamine, and non-essential amino acids (NEAA) were obtained from the Tissue Culture facility at Fox Chase Cancer Center. Cells were grown in DMEM supplemented with 10% FBS, 2 mM L-glutamine, NEAA, and P/S at 37°C under a 5% CO2 humidified atmosphere. Etoposide, aphidicolin (Aph), 5,6-dichloro-1-D-ribofuransylbenzimidazole (DRB), MG132, and n-propyl gallate were purchased from Sigma-Aldrich (MO). Glycerol, PIPES, EGTA, and formaldehyde were purchased from Fisher Scientific (PA). Mouse anti-RPA2 antibody was from Calbiochem (CA). Rabbit antibodies against CenpF, Top2α, and Top2β were kindly provided by Dr. Timothy Yen. Click-iT EdU Alexa Fluor 647 imaging kit with Hoechst, goat anti-mouse Alexa Fluor 488, and goat anti-rabbit Alexa Fluor 568 were purchased from Invitrogen (CA). Top2α siRNA (S102665068) and Top2β siRNA (S102780736) were purchased from Qiagen (MD). Control non-targeting siRNA (D-0012101-03) was purchased from Dharmacon (CA).

### Indirect immunofluorescence staining

U2OS cells were seeded in 24-well plates containing coverslips at a density of 8,000 cells per well. After two days of growth, cells were treated with etoposide and various inhibitors. Etoposide was added at the indicated concentrations to the media and then incubated for 2 hours. For pre-treatment with other inhibitors, Aph (30µM), DRB (300µM), and MG132 (20µM) were added 30 minutes prior to etoposide addition and were present throughout the subsequent etoposide treatment. To follow DNA synthesis, EdU was added 15 minutes prior to etoposide. For immunostaining, cells were pre-extracted with 0.1M PIPES(pH6.9)/1mM EGTA/4M glycerol/0.2% Triton X-100 for 1 minute, washed with 0.1M PIPES(pH6.9)/1mM EGTA/4M glycerol for 2 minutes, and fixed with 3.7% formaldehyde/50mM PIPES (pH6.9)/1mM MgCl_2_/5mM EGTA for 20 minutes. Cells were then incubated with primary antibodies against mouse anti-RPA2 and rabbit anti-CenpF followed by secondary antibodies goat anti-mouse Alexa Fluor 488 and goat anti-rabbit Alexa Fluor 568. For detection of EdU incorporation, cells were first stained with azide Alexa Fluor 647 following the manufacturer’s procedure before antibody staining. DNA was counterstained with Hoechst-containing mounting solution (1x PBS/4% n-propyl gallate/90% glycerol). Images were collected with a monochrome DAGE-MTI cooled CCD-300-RT camera under the control of Scion Image 1.6.1 (Scion Corp, MD) and processed for proper contrast/level and pseudo-colors in Photoshop CS 4.0 (Adobe Systems, CA).

### Data analysis

 At least 200 nuclei were counted and at least three sets of data were collected for each condition. The averages and standard deviations of the percentages of RPA focus positive nuclei were calculated and plotted. For comparisons of means, a two-tailed T-test was conducted at 95% confidence level (c.l.) unless otherwise indicated.

### Top2 depletion with siRNAs

U2OS cells were seeded in 24 well plates containing coverslips at a density of 6,000 cells per well. After 24 hours of incubation, cells were transfected with 20nM of the following siRNAs: Top2α, Top2β, Top2α + Top2β, or control. This was repeated after 24 hours and, after another 48 hours, cells were treated with etoposide for 2 hours and stained as described above.

## Results

### Low concentration etoposide induces RPA foci only in S phase cells

**Figure 1:**
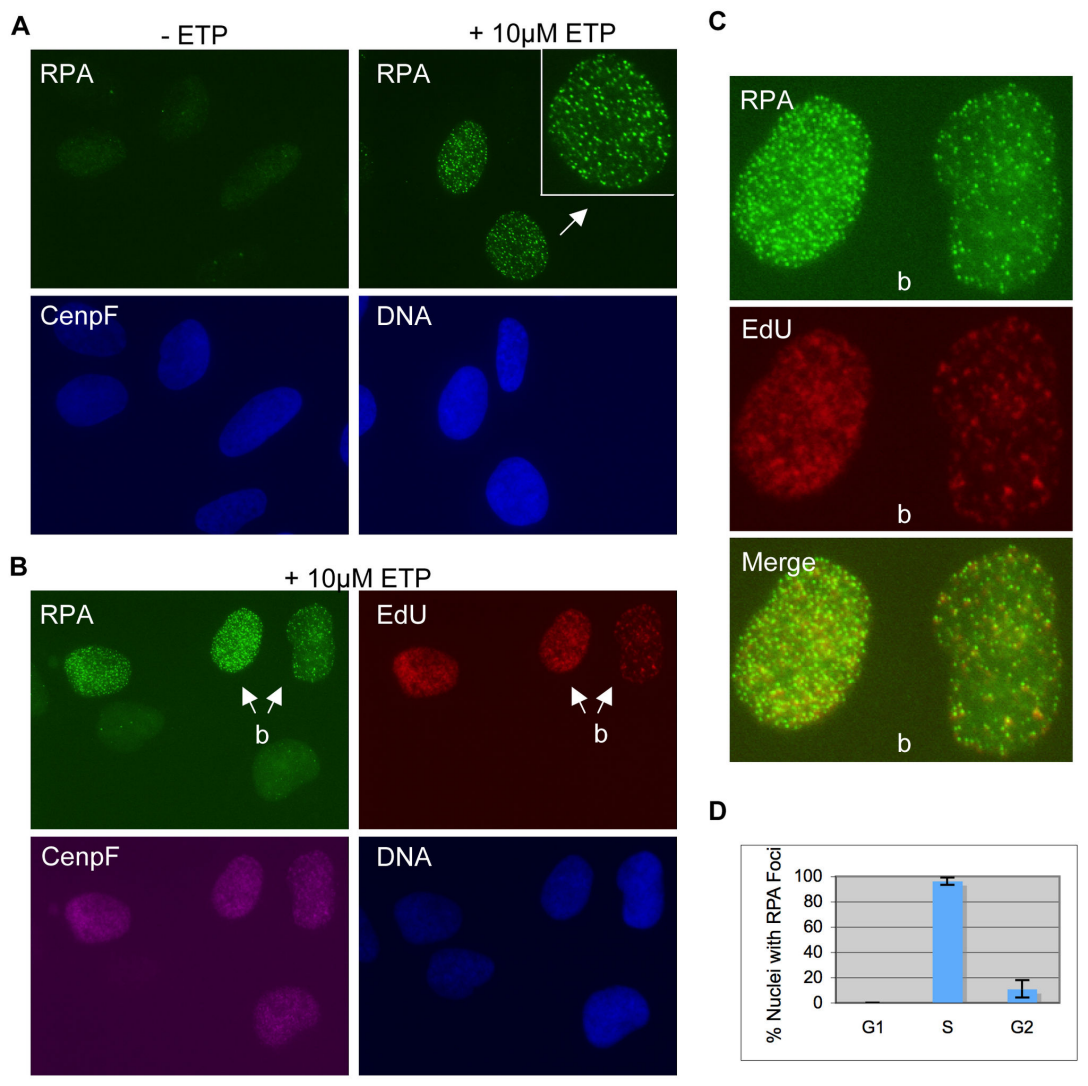
Etoposide at low concentrations induces RPA foci in S phase cells only. (**A**) U2OS cells were treated with or without 10µM etoposide for 2 hours and then fixed for staining with antibodies against RPA. DNA was stained with Hoechst. (**B**) U2OS cells were pre-incubated with EdU for 15 minutes before the addition of 10µM etoposide. After two further hours of incubation, cells were fixed and stained for RPA, EdU, and CenpF. DNA was stained with Hoechst. RPA foci were slightly reduced in intensity by the EdU staining protocol but remained discrete and clearly visible. Arrows indicate the two nuclei to be shown in enlarged format in (**C**). (**C**) Enlarged pictures of the nuclei indicated by the arrows in (**B**). (**D**) Percentages of RPA foci positive cells in each cell cycle stage were quantified and plotted.

Cells treated with etoposide developed a large number of discrete subnuclear foci of replication protein A (RPA), the eukaryotic single-stranded DNA binding protein (Figure **1A**). Compared to the weaker granular RPA staining in untreated cells, the etoposide-induced RPA foci are bright and discrete. Similar RPA foci have also been previously observed [36], and recent studies suggest that they represent the RPA molecules bound to 3’ ss-DNA resected from DSBs [37]. Resection is under cell cycle control and occurs during S and G2 phases when CDK2 activates the key resection protein CtIP via phosphorylation [38]. Consistent with this interpretation, etoposide-induced RPA foci were detected only in a subset of cells. To further demonstrate if these RPA foci positive cells are in S and G2 phases, we analyzed the relationship between RPA focus induction and cell cycle stage. Two markers were used to determine cell cycle stage: EdU, a nucleotide analog incorporated into DNA in S phase cells, and CenpF, a kinetochore protein that accumulates in S phase and peaks in G2 [39]. EdU was added 15 minutes before the addition of 10µM etoposide. After 2 hours of treatment with etoposide, cells were fixed and triple-stained for RPA, EdU, and CenpF. As shown in Figure **1B**, RPA foci were detected mainly in EdU^+^ nuclei (S phase), rarely in CenpF^+^ but EdU^-^ nuclei (G2), and completely absent in CenpF^-^ and EdU^-^ nuclei (G1) nuclei. Some nuclei showed limited DNA synthesis and their RPA foci were usually associated with regions of EdU staining (Figure **1C**). A quantitative analysis of RPA foci positive nuclei against cell cycle stage showed that while over 96% of S phase nuclei showed a large number of RPA foci, only 11% of G2 nuclei and 0% of G1 nuclei showed RPA foci (Figure **1D**). (The few RPA foci negative S phase cells were small in size and showed only weak EdU staining, suggesting that they were in early S phase and have no need for Top2 yet (data not shown)). Together, these observations suggest that 10µM etoposide induces RPA foci mainly in S phase. Since both S phase and G2 phase cells are able to resect DSBs, this result also indicates that 10µM etoposide can efficiently induce DSBs in S phase but not in G2 phase cells.

### RPA induction by low concentration etoposide is predominantly stimulated by replication rather than transcription

**Figure 2:**
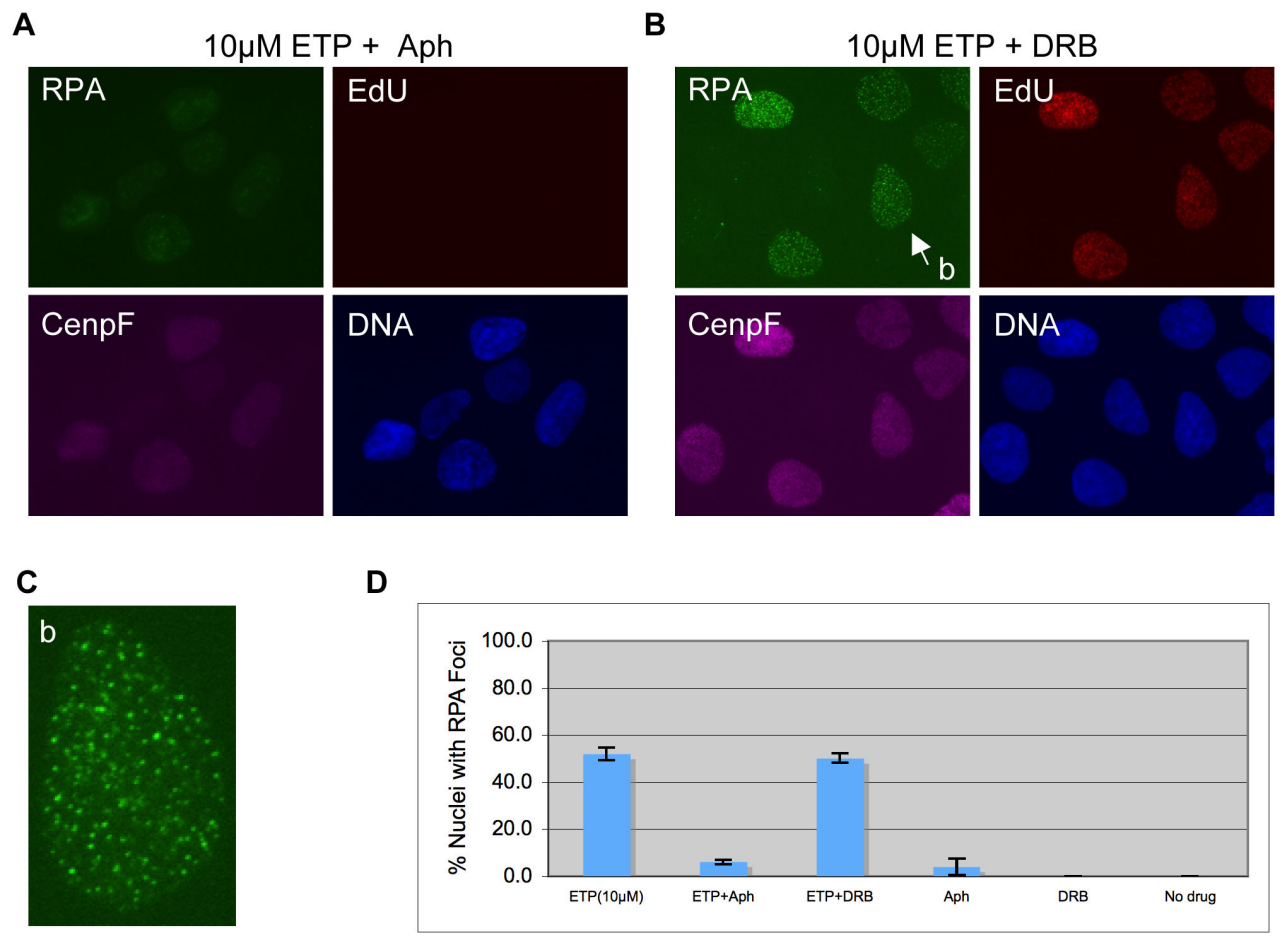
RPA focus induction by low concentration etoposide is dependent on replication but not on transcription. U2OS cells were pre-treated with aphidicolin (**A**) or DRB (**B**) for 30 minutes and then with 10µM etoposide for 2 hours. (EdU was added 15 minutes before etoposide). They were fixed, and stained for RPA, EdU, and CenpF. DNA was stained with Hoechst. (**C**) Enlarged picture of the nucleus indicated by the arrow in (**B**). (**D**) The percentages of RPA foci positive cells under each condition were quantified and plotted.

The above observation suggests that DSB induction by etoposide might be coupled to DNA replication. Conceivably, a replication fork might collide with a Top2cc, converting into a DSB. If so, blocking DNA synthesis should prevent RPA focus formation. We tested this hypothesis with aphidicolin, a specific inhibitor of replicative DNA polymerases and [40]. Briefly, aphidicolin was added 15 minutes before EdU (and 30 minutes before etoposide) to the media. After the addition of 10µM etoposide, cells were incubated for a further two hours before being fixed and stained for RPA, CenpF, and EdU. Under this condition, EdU incorporation into DNA was no longer detectable, and RPA focus formation was strongly inhibited (Figure **2A**). The number of nuclei with large numbers of discrete RPA foci was dramatically reduced from ca. 52% down to 6% (p=0), which is not statistically different from the 4% caused by aphidicolin alone (p=0.38) (Figure **2D**). This suggests that DSB induction by low concentration etoposide is the result of replication fork collision with Top2ccs.

This finding is somewhat unexpected as it has been shown previously that transcription rather than replication is the dominant process mediating the induction of DSBs by etoposide [28]. We thus examined how inhibiting transcription might impact RPA focus formation. Instead of aphidicolin, we pre-treated cells with 5,6-dichloro-1-D-ribofuransylbenzimidazole (DRB), an inhibitor of RNA polymerase II-dependent transcription that has been shown to block Top2 degradation and DSB formation induced by etoposide [28,41]. As shown in Figure **2B**, RPA foci still formed efficiently in the presence of DRB, albeit with a slight reduction in staining intensity. The percentage of nuclei with RPA foci was ca. 50% after DRB treatment, not significantly different from ca. 52% without DRB (p=0.38) (Figure **2D**). Together, these observations suggest that with 10µM etoposide, replication rather than transcription is the dominant process mediating the formation of DSB formation in cells. 

### High concentration etoposide induces RPA foci in both S and G2 cells and by both replication-dependent and transcription-dependent mechanisms

**Figure 3:**
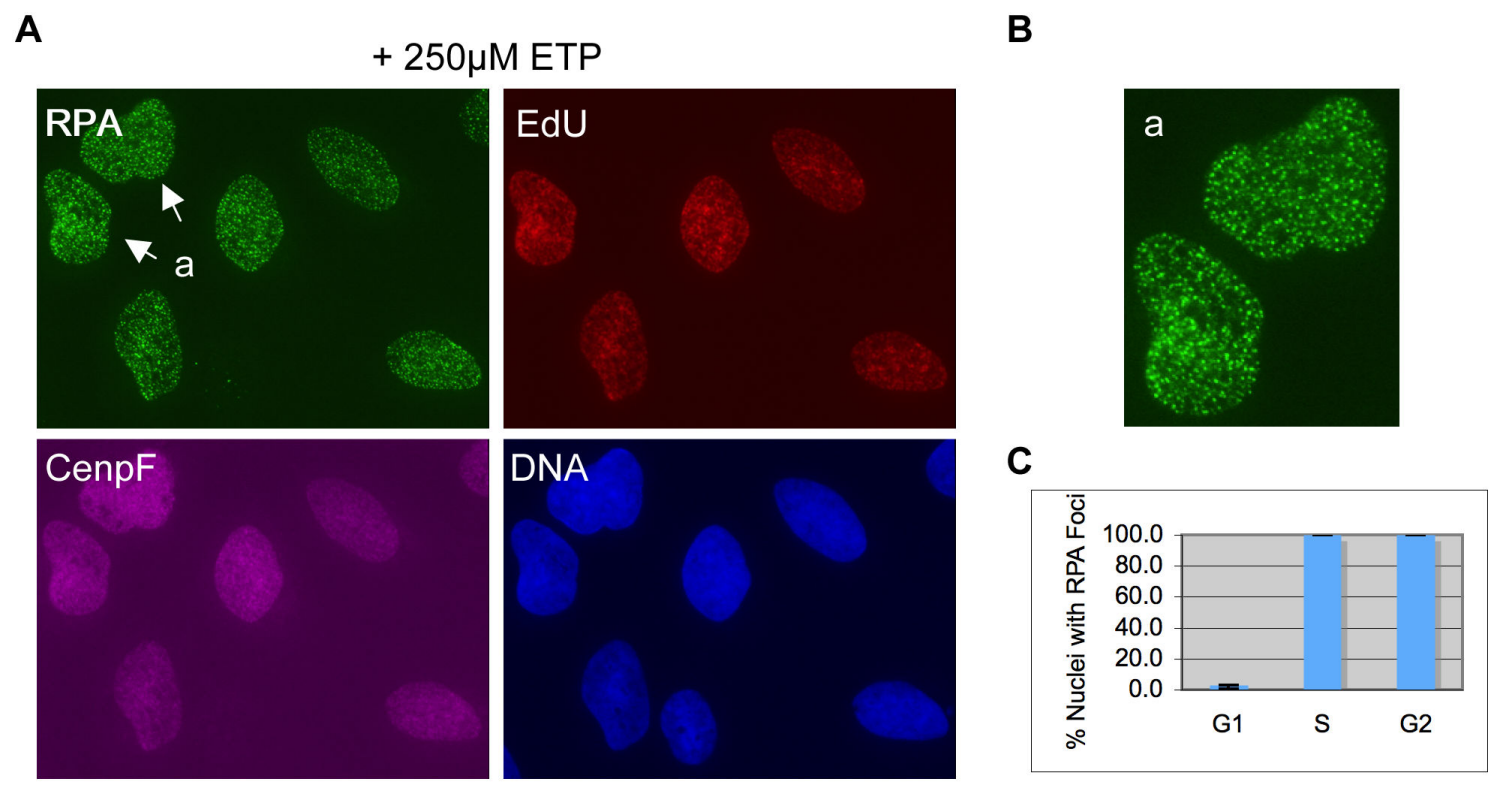
Etoposide at high concentrations induces RPA foci in S and G2 phase cells. (**A**) U2OS cells were pre-treated with EdU for 15 minutes and then with 250µM etoposide for 2 hours. They were fixed and stained for RPA, EdU, and CenpF. DNA was stained with Hoechst. (**B**) Enlarged picture of the nucleus indicated by the arrow in (**A**). (**C**) Percentages of RPA foci positive cells in each cell cycle stage were quantified and plotted.

Previous studies have suggested that etoposide induces DSBs by a transcription-dependent mechanism [28,30]. However, our data showed that this is not the case for 10µM etoposide. In the study that showed transcription as the dominant process mediating DSB formation, the concentration of etoposide used was 250µM, much higher than the 10µM used in our experiment described above [28,30]. To determine if this might account for the difference in the two studies, we examined the induction of RPA foci at 250µM etoposide. As shown in Figure **3A&B**, there was a massive induction of RPA foci at this concentration of etoposide. Co-staining with EdU and CenpF showed that not only S phase cells (EdU^+^) but also G2 phase cells (EdU^-^/CenpF^+^) formed discrete RPA foci. Even a small fraction of G1 cells (EdU^-^/CenpF^-^) showed some RPA foci, but the number of foci was low (

< 20) (presumably resulting from basal level resection in G1 cells). Quantitative analysis revealed that all S and G2 cells were positive for RPA foci (**Figure 3C**). This suggests that DSBs were formed in both S phase and G2 phase cells at high concentrations of etoposide.

**Figure 4:**
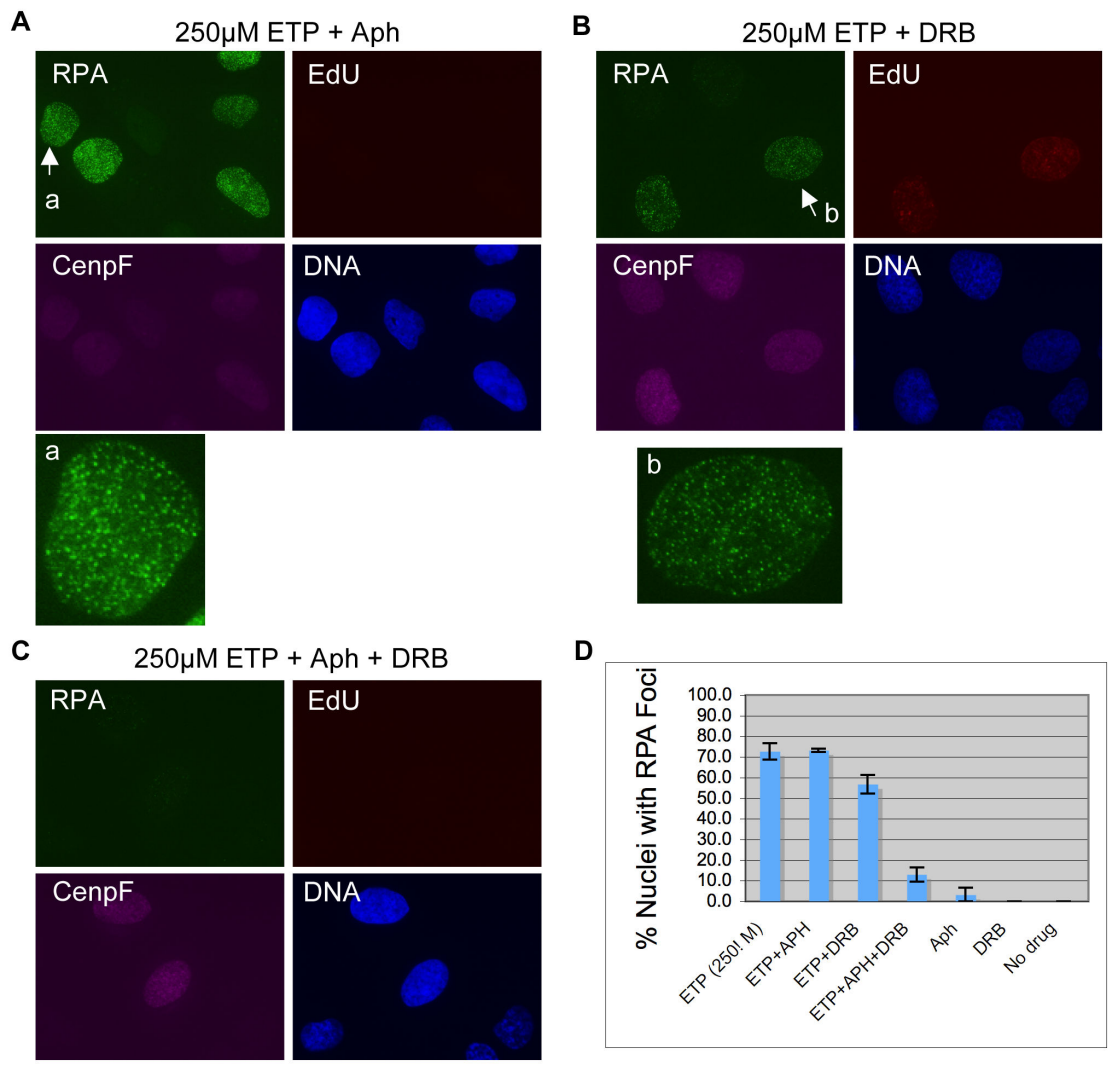
RPA focus induction by high concentration etoposide is dependent on replication and transcription. Cells were pre-treated with Aph (**A**), DRB (**B**), or both Aph and DRB (**C**) for 30 minutes and then with 250µM etoposide for 2 hours. (EdU was added 15 minutes before etoposide). They were then fixed, and stained for RPA, EdU, and CenpF. DNA was stained with Hoechst. The nuclei indicated by the arrows are also shown in enlarged format in the center panels. (**D**) The percentages of RPA foci positive cells under each condition were quantified and plotted.

 To determine how replication and transcription might impact RPA focus induction by high concentration etoposide, we then analyzed the effect of aphidicolin and DRB. As shown in Figure **4A**, in contrast to the effect on low concentration etoposide, aphidicolin did not significantly inhibit RPA focus induction by high concentration etoposide. RPA foci were still efficiently formed, and the percentage of positive cells was not altered (Figure **4D**). DRB did have some effect. RPA foci were slightly fainter and the percentage of foci positive nuclei was reduced from 72% to 57% (p=1.48E-4) (Figure **4D**). When both aphidicolin and DRB were used, the result was a dramatic reduction in RPA focus induction (Figure **4C**). The percentage of nuclei with significant RPA foci decreased from 72% down to 12% (p=0) (Figure **4D**). These observations suggest that at high concentrations of etoposide, both replication and transcription can mediate the induction of DSBs.

### Transcription-dependent but not replication-mediated RPA focus induction is sensitive to proteosome inhibitor MG132

**Figure 5:**
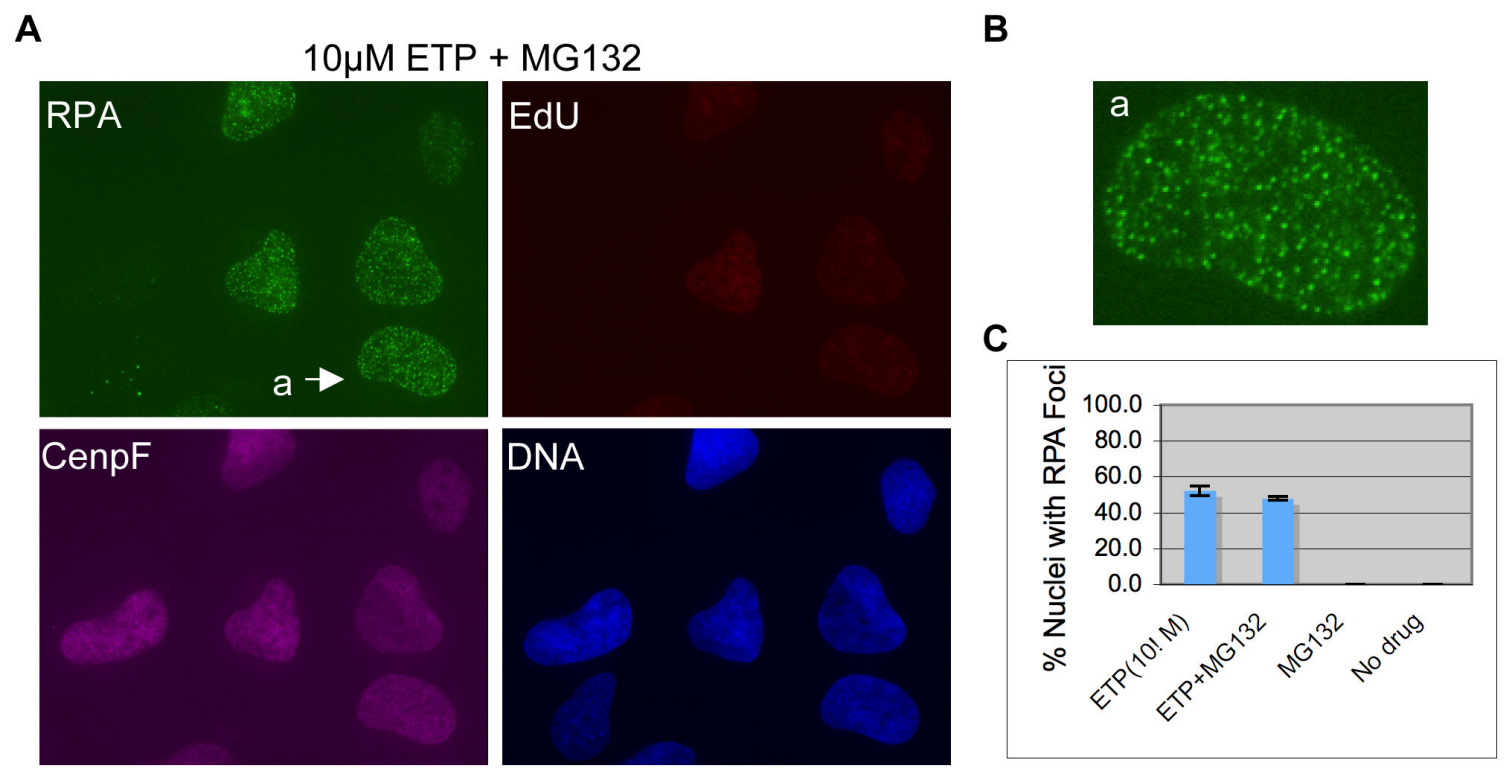
RPA focus induction by the replication-dependent mechanism does not require the 26S proteosome-mediated degradation of Top2cc. (**A**) MG132 was added 30 minutes before etoposide (and 15 minutes before EdU). Cells were fixed and stained for RPA, EdU, and CenpF. DNA was stained with Hoechst. (**B**) Enlarged picture of the nucleus as indicated by the arrow in (**A**). (**C**) Percentages of RPA foci positive cells under each condition were quantified and plotted.

**Figure 6:**
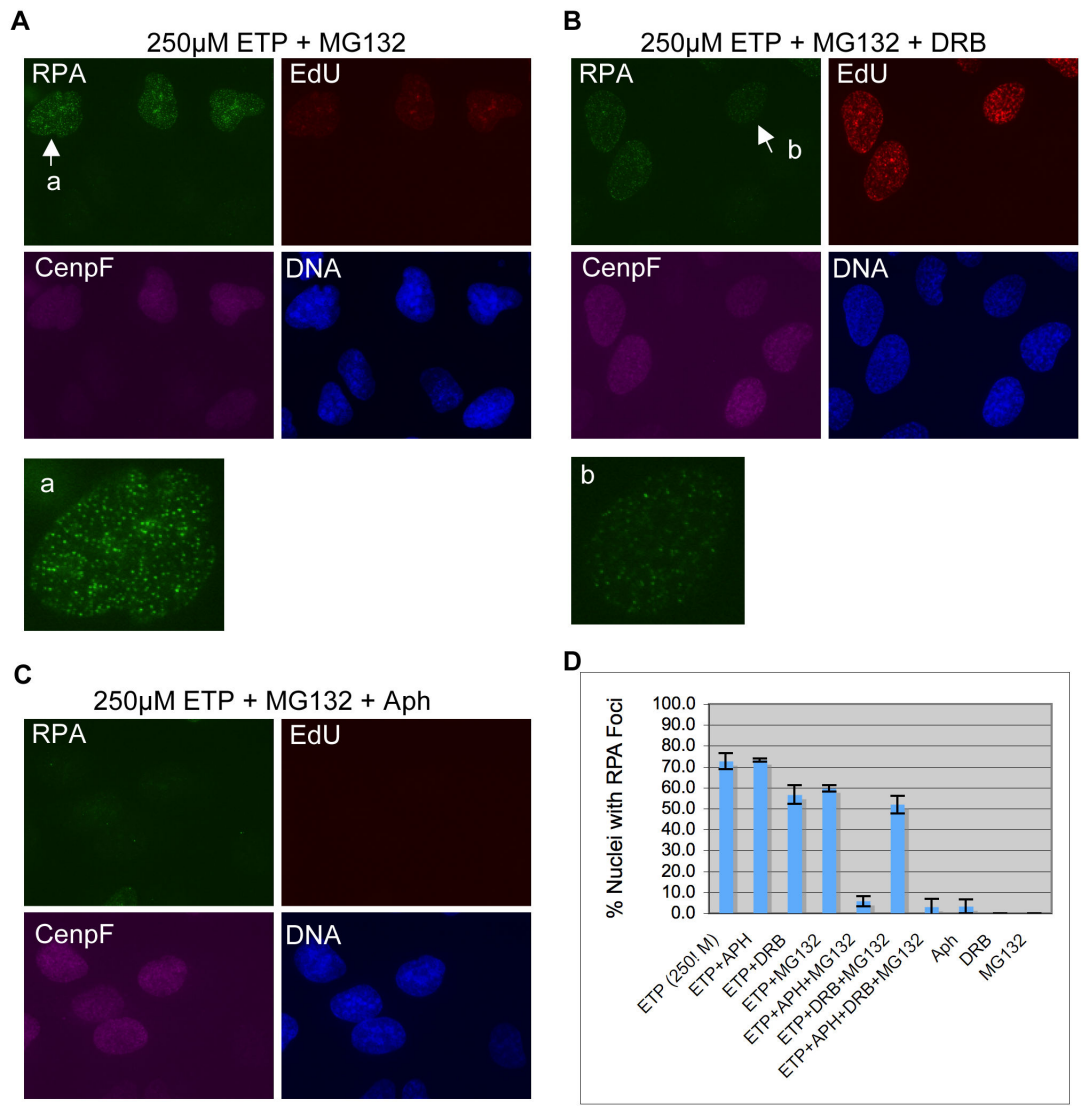
RPA focus induction by the transcription-dependent mechanism is dependent on the 26S proteosome-mediated degradation of Top2cc. Cells were pre-treated with MG132 (**A**), MG132 and Aph (**B**) or MG132 and DRB (**C**). They were then treated with 250µM etoposide for 2 hours, fixed, and stained for RPA, EdU, and CenpF. DNA was stained with Hoechst. The nuclei indicated by arrows are also shown in enlarged format in the center panels. (**D**) Percentages of RPA foci positive cells under each condition were quantified and plotted.

It has been shown that the transcription-dependent mechanism of DSB induction depends on the proteolysis of Top2cc by the 26S proteosome [28,30]. To determine if the replication-dependent mechanism is also dependent on proteolysis, we examined the effect of MG132, a specific inhibitor of the 26S proteosome, on RPA focus formation by etoposide at low and high concentrations. As shown in Figure **5A**, at 10µM etoposide, RPA foci still formed in the presence of MG132, albeit with a slight reduction in intensity. The percentage of nuclei with RPA foci was 48% with MG132, compared to 52% without (p=0.06) (Figure **5C**). At 250µM etoposide, MG132 did display a modest effect. RPA foci still formed efficiently, but the percentage of RPA foci positive nuclei was reduced from 72% down to 60% (p=9.8E-4) (Figure **6A&D**). Pre-treatment with both MG132 and DRB was similar to single pre-treatments of either drug, and the percentage of RPA foci positive nuclei was still over 52%, which is not significantly different from 57% for DRB alone (p=0.12) and 60% for MG132 alone (p=0.024; but not significant at 99% c.l.) (Figure **6B&D**). In contrast, when combined with aphidicolin, MG132 caused almost a complete inhibition of RPA focus induction. The percentage of RPA foci positive nuclei was reduced down to 5% (p=0) (Figure **6C&D**). Together, these results suggest that the 26S proteosome is indeed involved in RPA focus induction, but it acts in the same pathway as the transcription-dependent mechanism and is significant only at high concentrations of etoposide. The replication-dependent mechanism acts in a parallel pathway and does not require the 26S proteosome-mediated degradation of Top2cc.

### Top2α is the major isoform for the replication-dependent pathway while both Top2α and Top2β mediate the transcription-dependent pathway

**Figure 7:**
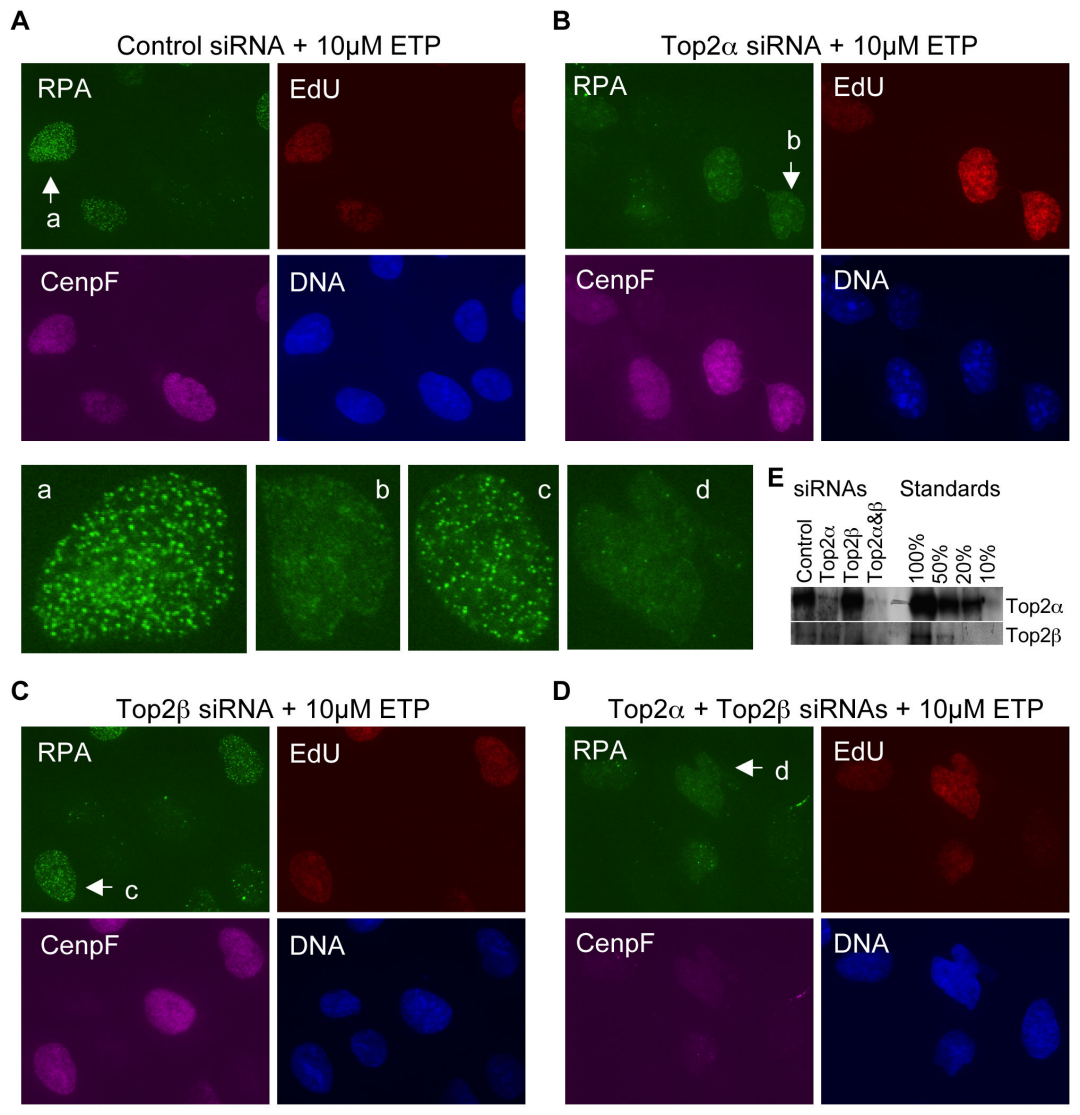
Top2α is the major isoform mediating the replication-dependent DSB induction mechanism. Cells were treated with two rounds of control siRNAs or Top2α and Top2β siRNAs for 72 hours. They were then treated with 10µM etoposide for 2 hours, fixed, and stained for RPA, EdU, and CenpF. DNA was stained with Hoechst. (**A**)-(**D**): siRNA treated cells stained for RPA, EdU, CenpF, and DNA. The nuclei indicated by arrows are also shown in enlarged format in the center panels. (**E**). Western blot analysis of Top2α and Top2β levels in siRNA treated cells. Different amounts of U2OS cell lysates were used as quantification standards.

Mammalian cells contain two isoforms of Top2, Top2α and Top2β, and both are targets of etoposide. The finding that there are two mechanisms for DSB induction raises an important mechanistic question: is a particular isoform responsible for a particular DSB induction mechanism? To address this question, we determined the respective roles of Top2α and Top2β in etoposide-induced RPA focus formation. Briefly, U2OS cells were treated with two rounds of siRNAs against Top2α and Top2β, alone or together, or with control siRNAs for a total of 72 hour. The levels of Top2α and Top2β were reduced to below detection with their respective siRNAs (Figure **7E**). Cells were then treated with etoposide at either low or high concentrations and finally fixed for staining for RPA, CenpF and EdU. As expected, knockdown of Top2α had a severe effect on chromosome segregation in many cells, as manifested by the chromosome bridges frequently present between two daughter cells (Figure **7B**). Most of the cells were in S or G2 phases, as indicated by the EdU and CenpF stainings. This is consistent with the observation that Top2α’s role in DNA replication is not for the synthesis of new strands but for the decatenation of replicating DNA. With 10µM etoposide, where the replication-dependent mechanism is dominant, Top2α knockdown caused a dramatic reduction in RPA focus formation (Figure **7B**). This was in contrast to the control siRNA treatment, where RPA foci were readily formed in S phase cells (Figure **7A**). Top2β knockdown had no discernable effect and the double knockdown of Top2α and Top2β was similar to the Top2α single knockdown (Figure **7C&D**).

**Figure 8:**
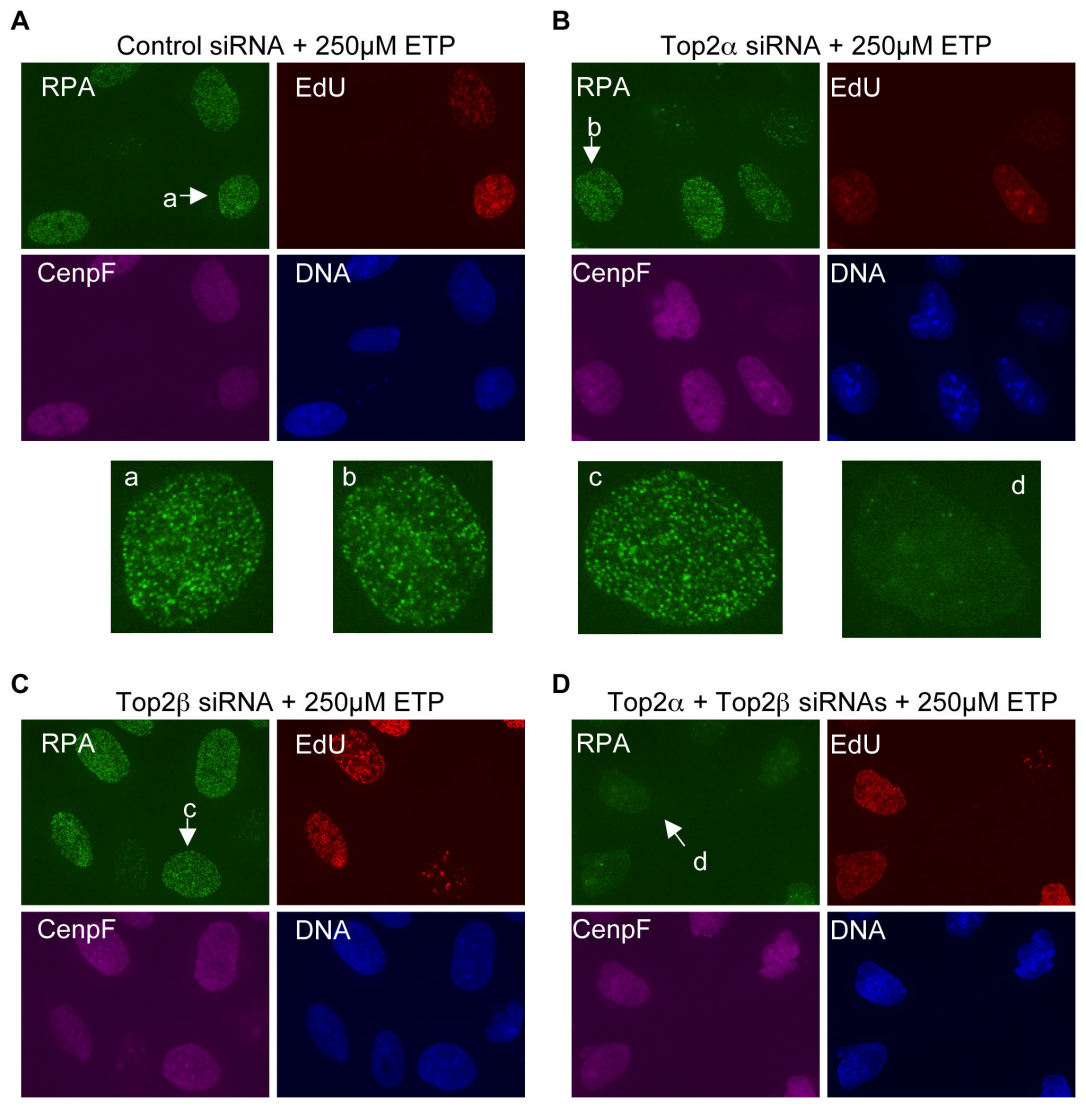
Top2α and Top2β can both mediate the transcription-dependent DSB induction mechanism. Cells were treated with two rounds of control siRNAs or Top2α and Top2β siRNAs for 72 hours. They were then treated with 250µM etoposide for 2 hours, fixed, and stained for RPA, EdU, and CenpF. DNA was stained with Hoechst. The nuclei indicated by arrows are also shown in enlarged format in the center panels.

With 250µM etoposide, when both the replication-dependent and the transcription-dependent mechanisms are active, the effect of Top2α knockdown on RPA focus induction was largely attenuated. RPA foci were still formed efficiently in the majority of S and G2 cells (Figure **8A&B**). Top2β knockdown again showed no discernable effect, even in G2 cells in which DSBs are induced by the transcription-dependent mechanism (Figure **8C**). When Top2α and Top2β were both knocked down, the induction of RPA foci was completely eliminated (Figure **8D**). Together, these results showed that RPA focus induction by etoposide is indeed mediated by both isoforms of Top2. Top2α is the major isoform for the replication-dependent DSB induction mechanism, but both Top2α and Top2β contribute to the transcription-dependent mechanism. The overall contribution of Top2α is greater than that of Top2β.

## Discussion

The major findings of this study are: (1) At low concentrations of etoposide, RPA foci are formed in S phase cells (2). At high concentrations of etoposide, RPA foci are formed in both S and G2 phase cells (3). At low concentrations of etoposide, RPA foci are induced mostly by a replication-dependent mechanism (4). At high concentrations of etoposide, RPA foci are induced by a replication-dependent mechanism and a transcription-dependent mechanism (5). The transcription-dependent mechanism requires proteolysis, but the replication-dependent mechanism does not (6). Top2α is the major isoform mediating DSB induction by etoposide and participates in both the replication-dependent mechanism and the transcription-dependent mechanism (7). Top2β mediates the transcription-dependent mechanism.

**Figure 9:**
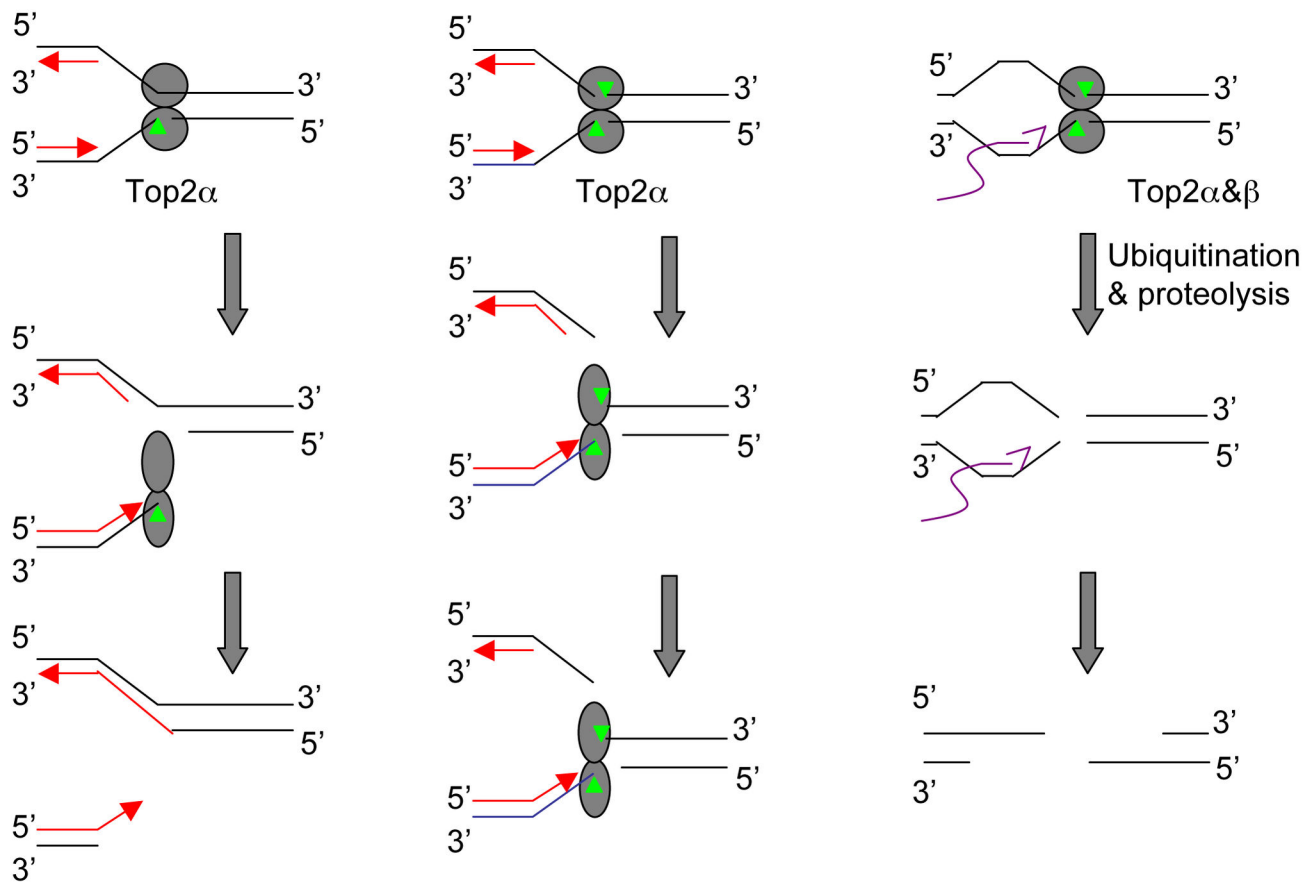
Model for DSB Induction by Etoposide in Cells. At low concentrations of etoposide, one subunit of Top2 is occupied by the drug to form a ss-Top2cc. Upon collision with the replication fork, ss-Top2cc is converted into a DSB, either directly by replication run-off (depicted) or indirectly by nucleolytic processing of collapsed replication forks (not depicted). At high concentrations of etoposide, both subunits of Top2 are occupied by the drug, resulting in a ds-Top2cc. It can be converted into a DSB by collision with the replication machinery or by degradation, which is stimulated by collision with the transcription machinery. Degradation might also occur independently of transcription.

What might these findings reveal about the mechanism of DSB induction by etoposide? Two key observations established by previous studies have to be considered. Firstly, none of the inhibitors, aphidicolin, DRB, or MG132, affect the formation of Top2ccs per se [41], so their effects on DSB induction are on the downstream cellular processing events. Secondly, etoposide acts as a two-drug, i.e., it binds independently to each of the Top2 subunits. Each drug molecule inhibits only the re-ligation reaction of the subunit it binds to, thus the resulting Top2cc can be either single-stranded (ss-Top2cc) or double-stranded (ds-Top2cc) depending on the concentration of etoposide [42]. In light of these observations, the findings of this study thus support the following model for the induction of DSBs by etoposide in cells (Figure **9**). The Ki of etoposide for Top2 is as high as 20µM [2]. At low concentrations of etoposide, the majority of DNA breaks are single-stranded [43,44]. Upon collision with the replication machinery, single-stranded Top2ccs (ss-Top2ccs) are converted into DSBs. How this conversion actually occurs is currently unknown. One potential mechanism is that the conformation of Top2ccs might be distorted upon collision with replication fork complexes, resulting in the release of the 3’ end to form DSBs (replication run-off). Alternatively, Top2ccs might lead to the stall and collapse of replication forks, which are then processed into DSBs by structure-specific nucleases such as Mus81 [45]. Neither of these two mechanisms requires Top2cc degradation. Collision with the transcription machinery can stimulate the degradation of ss-Top2ccs, but this would reveal single-strand breaks rather than DSBs. At high concentrations of etoposide, both subunits of Top2 are bound by etoposide and both strands of DNA are nicked and covalently linked to Top2 to form ds-Top2ccs. Ds-Top2ccs can still be converted into DSBs upon collision with the replication machinery, but they can now also be converted into DSBs by degradation mediated by the 26S proteosome. Collision with the transcription machinery strongly stimulates Top2cc degradation. As such, DSB induction at high concentrations of etoposide can be inhibited only if both replication and transcription are blocked. Top2α participates in DNA replication and its expression is up-regulated in S and G2 cells. It’s therefore the major mediator of the replication-dependent DSB induction mechanism. Top2β participates in transcription and can therefore mediate the transcription-dependent mechanism. However, Top2α can also mediate the transcription-dependent mechanism, making it the major isoform mediating DSB induction by etoposide.

 This model is supported by many findings reported in literature. It has been observed that the cytotoxicity of Top2-targeting drugs such as m-AMSA and etoposide can be partially suppressed by aphidicolin, but only at low drug concentrations [34,35]. This is consistent with our finding that the replication-dependent mechanism is more important at low concentrations than at high concentrations of etoposide. It has also been reported that both transcription and replication participate in the activation of cell cycle checkpoints in response to etoposide, but of different types [41]. This is consistent with our finding that both replication and transcription can convert Top2ccs to DSBs but by different mechanisms and thus likely yield different end structures that are sensed by different checkpoint proteins. Finally, it has been repeatedly shown that Top2α is the primary isoform mediating the cytotoxicity of etoposide [28,32]. This is easily explained by our finding that Top2α participates in both the replication-dependent and the transcription-dependent DSB induction. At low concentrations of etoposide, the replication-dependent mechanism, which is exclusively mediated by Top2α, is dominant. Also, Top2α expression is up-regulated in S phase and G2 phase cells, further accentuating the importance of Top2α in mediating etoposide’s cytotoxicity in proliferative cells. Clearly, the exact contribution of each isoform is also affected by the efficiency of their degradation, which varies in different types of cells [41]. In particular, Top2β is expected to be the major isoform mediating etoposide’s effect in non-proliferative cells.

The critical role of replication in DSB induction by etoposide as demonstrated in this study makes it a little surprising that it has not been previously discovered. Replication fork collision is known to convert the single-stranded 3’ phosphotyrosine-DNA topoisomerase 1 cleavage complex (Top1cc) that can be trapped by drugs like camptothecin, into DSBs [46]. There is no *a priori* reason against replication forks acting in a similar way to convert Top2ccs into DSBs. The most likely explanation is the difference in assays to detect DSBs used in our study and previous studies. DSBs are commonly detected by the neutral COMET assay or neutral CFGE (constant field gel electrophoresis) assay [28,30,44], both of which are based on the principle that broken genomic DNA strands in cells embedded in agarose have a faster mobility than intact genomic DNA during electrophoresis. The sensitivity of such assays is determined by the number of DSBs and the structure of DNA. If there are insufficient numbers of DSBs, the genomic DNA would still be too large to migrate into agarose. Furthermore, replicating DNA molecules usually carry branches or bubbles, structures known to dramatically slow down electrophoresis mobility [47,48]. In the COMET assays of such studies, very high concentrations of etoposide (250µM) were used, which are expected to generate a large number of DSBs by both transcription-dependent and replication-dependent mechanisms. DNA fragments generated by the replication-dependent DSBs are likely to carry branches or bubbles, the tail detected in the COMET assay thus represents mostly DSBs derived from the transcription-dependent mechanism. In our study, DSBs are detected by immunofluorescence staining of RPA foci, which represent 3’ ss-DNA derived from the resection of 5’ strands. Each DSB is expected to generate a RPA focus, making the assay extremely sensitive. It is un-affected by abnormal mobilities of structures like branches or bubbles, avoiding the complication of DNA replication intermediates as in the COMET assay. Importantly, in agreement with the COMET assay, it also revealed an important role for the transcription-stimulated degradation of Top2 in DSB induction by etoposide. This in turn provides strong validation for the RPA focus assay for the detection of DSBs. 

A limitation of this assay is that resection is activated in S and G2 cells and occurs only at DSBs channeled to HR but not those to NHEJ. However, the choice between NHEJ and HR is made after a DSB is formed. As such, in S and G2 cells, RPA staining reveals the formation of all DSBs rather than just those channeled to HR. In G1 cells, resection activity is low and NHEJ is the dominant DSB repair pathway. Previous studies using the COMET assay have shown that DSB induction by etoposide occurs in all stages of the cell cycle including G1 [32] and can be inhibited by DRB and MG132 [28,30]. In our studies, the basal level RPA foci induced by high concentrations of etoposide in G1 cells are further reduced by DRB and MG132. Taken together, it is reasonable to conclude that G1 cells use the transcription-stimulated proteolysis of Top2ccs to generate DSBs. 

The findings of this study have strong implications for the optimal use of etoposide in cancer therapy. Current therapeutic regimens achieve plasma levels ranging from 10µM to 130µM [49]. Our finding that low concentration etoposide induces DSBs by a replication-dependent mechanism while high concentration etoposide does so by both replication-dependent and transcription-dependent mechanisms has important implications for the optimization of etoposide regimens. Continuous or frequent administration of low dose should have better efficacy by preferentially killing tumor cells, which contain high fractions of S phase cells, without inflicting significant damage to normal tissues such as heart, which are composed mostly of non-replicating cells. In contrast, a few large doses of etoposide might inflict too much damage to normal tissues without the extra benefit of more efficient killing of highly proliferative tumor cells. In addition, since Top2β appears to be the major isoform mediating etoposide-induced chromosomal translocations [30,50], lower drug doses, which are ineffective at converting Top2βccs to DSBs, might reduce the risk of secondary malignancies. Another implication of this study is that the end structures of the DSBs generated by the two mechanisms might be different. In the case of the transcription-dependent mechanism, the resulting ends are expected to carry a short peptide or 5’ phosphotyrosine. In the case of the replication-dependent mechanism, the resulting ends are likely to be either naked or carry a protein adduct. Further studies are required to understand how these different types of ends are repaired. Inhibiting specifically the DSBs generated by the replication-dependent mechanism might provide further improvement to the efficacy while reducing the side effects of etoposide in cancer therapy.
